# Prevalence, awareness, treatment and control of dyslipidemia among adults in Northwestern China: the cardiovascular risk survey

**DOI:** 10.1186/1476-511X-13-4

**Published:** 2014-01-06

**Authors:** Jun-yi Luo, Yi-Tong Ma, Zi-xiang Yu, Yi-Ning Yang, Xiang Xie, Xiang Ma, Fen Liu, Xiao-mei Li, Bang-dang Chen

**Affiliations:** 1 Department of Cardiology, First Affiliated Hospital of Xinjiang Medical University, Urumqi, Xinjiang, China; 2 Xinjiang Key Laboratory of Cardiovascular Disease Research, Urumqi, Xinjiang, China

**Keywords:** Dyslipidemia, Prevalence, Awareness, Treatment, Control

## Abstract

**Aim:**

The aim of this study was to estimate the prevalence, awareness, treatment, and control of dyslipidemia in Xinjiang, China.

**Method:**

Stratified sampling method was used to select a representative sample of the general population including Chinese Han, Uygur, and Kazak in this geographic area. Seven cities were chosen. Based on the government records of registered residences, one participant was randomly selected from each household. The eligibility criterion for the study was ≥ 35 years of age.

**Results:**

A total of 14,618 participants (5,757 Han, 4,767 Uygur, and 4,094 Kazak), were randomly selected from 26 villages in 7 cities. The prevalence of dyslipidemia was 52.72% in the all participants. The prevalence of dyslipidemia was higher in Han than that in the other two ethnic (58.58% in Han, 48.27% in Uygur, and 49.60% in Kazak, *P* < 0.000). The prevalence of dyslipidemia was higher in men than that in women (56.4% vs. 49.3%, *P* < 0.000). Among the participants with dyslipidemia, the proportion of those who aware, treat, control of dyslipidemia were 53.67%, 22.51%, 17.09% in Han, 42.19%, 27.78%, 16.20% in Uygur, 37.02%, 21.11%, 17.77% in Kazak.

**Conclusion:**

Dyslipidemia is highly prevalent in Xinjiang. The proportion of participants with dyslipidemia who were aware, treated, and controlled is unacceptably low. These results underscore the urgent need to develop national strategies to improve the prevention, detection, and treatment of dyslipidemia in Xinjiang.

## Introduction

Cardiovascular disease (CVD) is one of the first leading contributor to mortality both in developed and developing countries [1]. It has been estimated that aging and population growth will increase CVD by more than a half over the next 20 years in China [2]. This will bring such a enormous burden for Chinese economy. Dyslipidemia, an independent and modifiable risk factor has a causal role in the pathogenesis of CVD [3]. With the economic growth and associated lifestyle changes, studies demonstrated that the prevalence of dyslipidemia has increased significantly during the last decade, however the awareness, treatment and control of dyslipidemia was low in China [4-6]. These above studies mainly enrolled Chinese Han participants. The condition of dyslipidemia in minorities of China was incompletely investigated.

Xinjiang is an autonomous minority ethnic region of the People’s Republic of China in the northwest of the country. It is the largest Chinese administrative division and spans over 1.6 million km^2^ which takes up about one sixth of the country’s territory. The Tianshan mountains divide Xinjiang into two parts, the north is called the northern Xinjiang, the south is called the southern Xinjiang. Several studies had reported the prevalence, awareness, treatment and control of dyslipidemia among adults in Xinjiang [7-9]. Nevertheless, the samples in each study were small or the participants were from the northern or the southern area only. So, those studies couldn’t represent the status of dyslipidemia in the whole region of Xinjiang. To better understand the status of dyslipidemia in Xinjiang, our team did a cross-sectional study called the Cardiovascular Risk Survey (CRS) between October 2007 and March 2010, which was expected to provide a comprehensive update results for the previous studies.

## Methods

### Ethnic statement

This study was conducted according to the standards of the Declaration of Helsinki. Written informed consent was acquired from each participant prior to enrollment. Ethics approval was obtained from the Ethics Committee of the First Affiliated Hospital of Xinjiang Medical University.

### Study subjects

A multi-ethnic, community-based, cross-sectional study involving cardiovascular risk factors (called CRS) was conducted in Xinjiang, northwestern China between October 2007 and March 2010. The study has been described in detail previously [10-13]. Briefly, The CRS was designed to determine the prevalence, incidence, and risk factors of CVD and to determine the genetic and environmental contributions to atherosclerosis, coronary artery disease, and cerebral infarction in the population of Xinjiang, northwestern China. There were approximate 19.63 million residents lived in the beautiful place. Among them the Uygur accounted for 45.73% of the population, the Han accounts for 39.75% of the population, and the Kazak accounted for 7.04% of the population (data from 5^th^ census of China). We used a stratified sampling method to select a representative sample of the general population of the three main ethnics (Han, Uygur, and Kazak) residing in Xinjiang. Seven cities [northern Xinjiang (Urumqi, Karamay, Yili and Fukang), southern Xinjiang (Khotan, Turpan, and Shanshan)] were selected. Based on the government records of registered residences, one participant was randomly selected from each household. The eligibility criterion for the study was ≥ 35 years of age. A total of 14,618 participants (5,757 Han, 4,767 Uygur, and 4,094 Kazak) were randomly selected from 26 villages of the 7 cities and were invited to participate in our study. Participants with incomplete data were excluded, thus 14, 113 participants (5,582 Han, 4,616 Uygur, and 3,915 Kazak) were analyzed in this study.

### Data collection

#### Questionnaire interview

Data were collected in examination centers at the local hospitals in the participants’ residential areas. During the clinic or home visit, trained researchers asked the participants questions from a standard questionnaire providing information on demographic variables, such as age, gender, ethnicity, address, education, occupation, family income, a known diagnosis of dyslipidemia, and current treatment of dyslipidemia.

#### Laboratory assay

During the interview, a 5-mL fasting blood sample (fasting ≥ 8 hours) was collected in an EDTA vacutainer tube. Serum was separated from the samples within 30 min and stored at –80°C immediately after processing. We measured the concentrations of triglycerides (TG), total cholesterol (TC), and high density lipoprotein-cholesterol (HDL-C), and low density lipoprotein-cholesterol (LDL-C) using a Dimension AR/AVL Clinical Chemistry System (Newark, NJ, USA) in the Clinical Laboratory Department of the First Affiliated Hospital of Xinjiang Medical University.

#### Definitions

The definition of dyslipidemia was based on the guideline of Chinese Prevention and Treatment of Dyslipidemia in adults [14]. Thus, a subject was considered as dyslipidemia if his total cholesterol ≥ 6.22 mmol/l (240 mg/dl) and/or his LDL-C ≥ 4.14 mmol/l (160 mg/dl) and/or HDL-C < 1.04 mmol/l (40 mg/dl), and/or triglyceride ≥ 2.26 mmol/l (200 mg/dl) and/or he was taking a hypolipidemia drug. Awareness of dyslipidemia was defined as a self-report of any prior diagnosis of dyslipidemia by a medical doctor. Treatment of dyslipidemia was defined as use of pharmacological treatment to manage dyslipidemia during the previous 2 weeks. Participants were considered to have controlled dyslipidemia if their serum concentrate of TC < 6.22 mmol/l, LDL-C < 1.14 mmol/l, HDL-C ≥ 1.04 mmol/l, and TG < 2.26 mmol/l after treatment.

### Statistical analysis

All of the questionnaire data were double-entered and cross-validated using EpiData version 3.1 (EpiData Association, Odense, Denmark). Data analyses were conducted using a commercially available statistical software package (SPSS for Windows, version 17.0, SPSS, Chicago, IL). We stratified our analyses by age, sex, and ethnicity (Han, Uygur, and Kazak). Continuous variables were given as mean ± standard deviation and the categorical variables were given as percentages. One-way ANOVA was used to compare the means in numerical data. *χ*^2^-test was used to explore associations in categorical data. Logistic regression analysis was used to assess the contribution of smoking and alcohol to the prevalence of dyslipidemia. All *P*-value were two-side and significant difference was set at *P*-values < 0.05.

## Results

### Participant characteristics

The participants included in these analyses, 39.55% were Han, 32.71% were Uygur, and 27.74% were Kazak. The general characteristics of the participants are shown in Table 1.

**Table 1:** Participation characteristics in adult population , aged ≥ 35 years, in Xinjiang

	**Mean ± SD or No. (%)**		
	**Han**	**Uygur**	**Kazak**
	**(n = 5582)**	**(n = 4616)**	**(n = 3915)**
Age, years	52.6 ± 12.7	50.7 ± 13.0	48.7 ± 11.6
Height, cm	163.2 ± 8.5	159.8 ± 8.5	162.7 ± 8.7
Weight, kg	67.2 ± 12.0	66.1 ± 12.9	70.6 ± 15.1
Women, n (%)	2889 (51.8)	2669 (57.8)	2021 (51.6)
Smoking, n (%)	1749 (31.4)	871 (18.9)	1396 (35.7)
Alcohol, n (%)	1057 (18.9)	456 (9.9)	571 (14.6)
BMI, kg/m^2^	25.1 ± 3.5	25.8 ± 4.4	26.6 ± 4.8
SBP, mmHg	132.9 ± 19.9	131.5 ± 21.2	140.4 ± 25.1
DBP, mmHg	85.1 ± 15.5	80.1 ± 14.9	88.5 ± 19.7
Waist circumference, cm	86.8 ± 10.3	88.2 ± 12.3	88.3 ± 13.4
Hip circumference, cm	95.8 ± 7.4	97.7 ± 10.0	99.6 ± 9.7
TG, (mmol/L)	1.7 ± 1.4	1.6 ± 1.2	1.2 ± 0.9
TC, (mmol/L)	4.7 ± 1.1	4.4 ± 1.1	4.8 ± 1.2
HDL-C, (mmol/L)	1.3 ± 0.5	1.3 ± 0.5	1.3 ± 0.4
LDL-C, (mmol/L)	2.9 ± 0.9	2.9 ± 0.9	2.9 ± 0.9
Blood Glucose, (mmol/L)	5.3 ± 1.8	4.9 ± 1.7	5.1 ± 1.5

Note: *BMI*, body mass index, *SBP*, systolic blood pressure, *DBP*, diastolic blood pressure, *TG*, triglycerides, *TC*, total cholesterol, *HDL-C*, high density lipoprotein-cholesterol, *LDL-C*, low density lipoprotein-cholesterol.

### Prevalence of dyslipidemia

Table 2 shows the prevalence of dyslipemia of the participations. The prevalence of dyslipidemia was 52.72% in the total participants. The prevalence of dyslipidemia was significant higher in Han than that in the other two ethnic (*P* < 0.000). The prevalence of dyslipidemia was higher in men than that in women (*P* < 0.000). For total and women, the prevalence of dyslipidemia was significantly lower in the 35-54 years old group than that in 55-88 years old group among the three ethnic. For men, the prevalence of dyslipidemia was significantly higher in the 35-54 years old group than that in 55-88 years old group in the Han and Uygur ethnic. But for Kazak ethnic, no significant difference was found between the two age groups.

**Table 2:** Prevalence of dyslipidemia in adult population , aged ≥ 35 years, in Xinjiang

**Age, year**	**Men**		**Women**		**Total**	
	**N**	**Dyslipidemia [n, (%)]**	**N**	**Dyslipidemia [n, (%)]**	**N**	**Dyslipidemia [n, (%)]**
Han ethnic						
35-54	1628	1116 (68.6)	1641	681 (41.5)	3260	1797 (55.1)
55-88	1065	637 (59.8)	1248	836 (67.0)	2313	1473 (63.7)
Total	2693	1753 (65.1)	2889	1517 (52.5)	5582	3270 (58.6)
*P* value		<0.001		<0.001		<0.001
Uygur ethnic						
35-54	1071	529 (49.4)	1761	787 (44.7)	2832	1316 (46.5)
55-88	876	384 (43.8)	908	528 (58.2)	1784	912 (51.1)
Total	1974	913 (46.3)	2669	1315 (49.3)	4616	2228 (48.3)
*P* value		0.015		<0.001		0.002
Kazak ethnic						
35-54	1287	699 (54.3)	1450	571 (39.4)	2737	1270 (46.4)
55-88	607	336 (55.4)	571	336 (58.8)	1178	672 (57.1)
Total	1894	1035 (54.7)	2021	907 (44.9)	3915	1942 (49.6)
*P* value		0.671		<0.001		<0.001

### Smoking and drinking related to the prevalence of dyslipidemia

The Table 3 shows that the prevalence of dyslipidemia was significantly related to smoking (ex-smoker and current smoker have significantly higher risk for dyslipidemia than that the person who never smoked), alcohol consumption (current drinker has significantly higher risk for dyslipidemia than that the person who never drunk).

**Table 3:** smoking and alcohol consumption to the dyslipidemia in Xinjiang area

**Risk factors**	**OR**	**Lower of 95.0% CI**	**Upper of 95.0% CI**	***P *value**
Smoking status				
Ex-smoker	2.2	2.0	2.3	*P* < 0.001
Current smoker	4.0	3.6	4.3	*P* < 0.001
Alcohol status				
Ex-drinker	1.1	0.9	1.2	0.405
Current drinker	1.3	1.2	1.4	*P* < 0.001

### Awareness, treatment, and control of dyslipidemia

**Figure 1:**
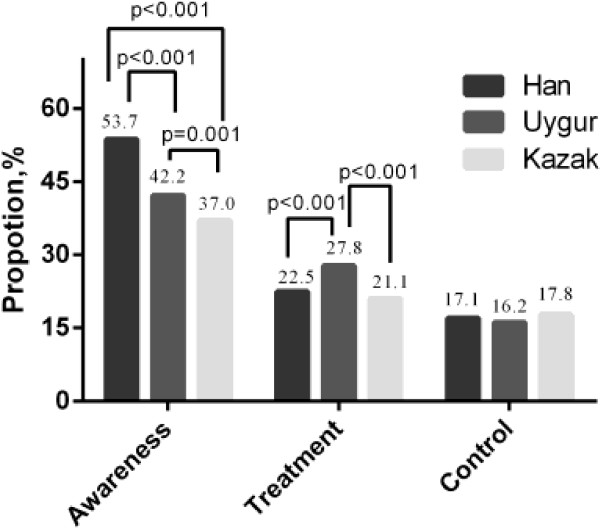
The percentage of awareness, treatment, control of dyslipidemia among the three ethnics in Xinjiang.

Among the participants who with dyslipidemia, the proportion of those who were aware of their condition was highest in Han than the other two ethnics (*P* < 0.001). The percentage of participants who was treated for dyslipidemia among the three ethnics was higher Uygur than that in Han or Kazak (*P* < 0.001). For the percentage of control of dyslipidemia, no significant difference was found among the three ethnics (Figure 1).

Table 4 shows the percentage of awareness, treatment, and control of dyslipidemia in the subgroups classified by age and sex in each ethnic. In Han, men of the younger age group (ages 35-54) had a significantly higher awareness of dyslipidemia than that in younger age women (58.96% vs. 49.05%, *P*<0.001). Nevertheless, the younger men had a significantly lower rate of control of dyslipidemia than that in women (13.80% vs. 19.53%, *P =* 0.001). Additionally, men of the older age group (ages 55-88) had a significantly lower treatment of dyslipidemia than that in older age women (22.92% vs. 27.75%, *P* = 0.035). However, the older men had a significantly higher rate of control of dyslipidemia than that in older women (21.35% vs. 16.27%, *P* = 0.013). Significant differences of the proportion of awareness (58.96% vs.53.38% *P* = 0.023) and control (13.8% vs. 21.35%, *P*<0.001) were found between the younger age group and the older age group in men. In addition, a significant difference of treatment of dyslipidemia was also found between the two age group in women (20.85% for younger group, 27.75% for older group, *P* = 0.002).

**Table 4:** Awareness, treatment, control of dyslipidemia in adult population , aged ≥ 35 years, in Xinjiang

**Age, year**	**Han [n, (%)]**		***P *value**	**Uygur [n, (%)]**		***P *value**	**Kazak [n, (%)]**		***P *value**
	**Men**	**Women**		**Men**	**Women**		**Men**	**Women**	
Awareness									
35-54	658 (59.0)	334 (49.1)	<0.001	216 (40.8)	336 (42.7)	0.502	269 (38.5)	204 (35.7)	0.312
55-88	340 (53.4)	423 (50.6)	0.291	164 (42.7)	224 (42.4)	0.932	133 (39.6)	113 (33.6)	0.109
*P* value	0.023	0.547		0.057	0.923		0.734	0.523	
Treatment									
35-54	216 (19.4)	142 (20.9)	0.441	106 (20.0)	234 (29.7)	<0.001	149 (21.3)	108 (18.9)	0.289
55-88	146 (22.9)	232 (27.8)	0.035	111 (28.9)	168 (31.8)	0.346	85 (25.3)	68 (20.2)	0.118
*P* value	0.076	0.002		0.02	0.421		0.152	0.626	
Control									
35-54	154 (13.8)	133 (19.5)	0.001	80 (15.1)	127 (16.1)	0.62	134 (19.2)	106 (18.6)	0.784
55-88	136 (21.4)	136 (16.3)	0.013	75 (19.5)	79 (15.0)	0.069	61 (18.2)	44 (13.1)	0.071
*P* value	<0.001	0.098		0.08	0.565		0.696	0.032	

In Uygur, no significant differences of awareness or control of dyslipidemia was found in each subgroup. Nevertheless, the younger age women had a significantly higher treatment of dyslipidemia than that in younger age men (29.73% vs. 20.04%, *P*<0.001). A significant difference of treatment of dyslipidemia was also found between the two age group in men (20.04% for younger group, 28.91% for older group, *P* = 0.02). In Kazak, we only found that the younger age women had a significantly higher control of dyslipidemia than that in the older age women (18.56% vs. 13.10%, *P* = 0.032).

## Discussion

The present study was aimed to determine the prevalence rate of dyslipidemia, and the awareness, treatment, and control of dyslipidemia in Xinjiang (northwestern China). We found that the prevalence of dyslipidemia was high in this area. The Chinese national nutrition and health survey (CNHS) indicated that the prevalence of dyslipidemia was 18.6% in the Chinese national average in 2002 [15,16]. Wu et al. reported that the prevalence of dyslipidemia of the population in Shanghai was 36.5% in 2003 [5]. Studies reported that the prevalence of dyslipidemia in the Beijing adult population was 30.3% in 2007 and 35.4% in 2008 [4,17]. It was reported that economic growth and associated changes in lifestyle and diet might contribute to the increase of the prevalence of dyslipidemia in the Chinese population. However, it was obvious that the prevalence of dyslipidemia in the three ethnic in Xinjiang were significantly higher than that in the other areas of China even the economically developed areas in China. This may be associated with the residents in Xinjiang were characterized by eating more animal fat, drinking strong wine, with a higher salt intake (>20 g per day), and consuming less grain, fresh vegetables, beans, bean products, and unsaturated fatty acids [18]. A significant difference of the prevalence of dyslipidemia was also observed among the subgroups classified by the sex and the age. The prevalence was higher in men that in women among participants under age 55, but the results were reversed in the participants older than 55 years. The phenomenon was consistent with the results of the Wu et al. [5]. The increased prevalence among older women may be associated to differences in estrogen levels between pre- and post-menopausal women [19].

In Xinjiang area, higher levels of dyslipidemia was found among the smokers (ex and current) and current drinkers. Those results are in agreement with other sources [20-22], and indicate the clustering of common cardiovascular risk factors within these populations.

We also observed that the prevalence of dyslipidemia in the Han ethnic was significantly higher than that in the Uygur and Kazak ethnic, the mechanisms underlying this phenomenon are not clear. It is believed that the different dietary habits among the three ethnics may play an important role [23]. The Han population consisted mainly of animal fats, such as pork, and the principal food was carbohydrate, especially refined flour or rice, whereas the Uygur and Kazak people subsist chiefly on beef, mutton, and milk products. Furthermore, the Uygur and Kazak population were characterized by eating onion, tea, yogurt, and nuts, which can promote lipid absorption, digestion, and decomposition [24]. Moreover, most of the Uygur and Kazak participations lived in cold and semiarid regions, they need more energy to cope with the cold whether than the Han population who most of them lived in the warm and plain regions. In addition, different of genetic backgrounds and gene-environment interactions might also be important factors underlying the different prevalence of dyslipidemia among the three ethnic.

The prevalence of dyslipidemia in the Xinjiang area was surprisingly high, but the rate of awareness, treatment, and control of dyslipidemia was significantly low in the participations based on the present study. For Han ethnic, the percentage of awareness, treatment, and control of participations with dyslipidemia was 53.67%, 22.51%, and 17.09%, respectively. For Uygur ethnic, the percentage was 42.19%, 27.78%, 16.20%, and for Kazak ethnic, the percentage was 37.02%, 21.11%, 17.77%. We found that the awareness and treatment of dyslipidemia were significantly higher in our study than that in the previous studies [4,6,25]. However, the control of dyslipidemia in our study was unacceptably low among the all participants who with dyslipidemia. It meant that the dyslipidemia has become one of the important health risk factors in the Xinjiang. Therefore, a national dsylipidemia education program that to promote community- and clinic-based serum lipid screening was urgently needed. Physicians must regularly check their patients’ serum lipids, and a more aggressive serum lipid-lowering goal and strategy must be utilized.

Our study was a large, comprehensive epidemiologic evaluation of ethnic and sex difference in dyslipidemia, but it did have some limitations. Firstly, the CRS was a cross-sectional study, potential recall bias of self-reported might affect the condition of dyslipidemia in Xinjiang. Secondly, our study enrolled the three main ethnics (Han, Uygur, Kazak) which consisted more than 90 percentage of the population in Xinjiang. Nevertheless, there were nearly more than 10 ethnic minorities lived in Xinjiang generation by generation, the status of dyslipidemia was still unclear.

## Conclusion

In conclusion, our study provides reliable and up-to-date information on dyslipdemia in the adult of Xinjiang. More than half of the participants had dyslipidemia in Xinjiang, it was significantly higher than the Chinese national average rate. But the proportion of awareness, treatment, and control was unacceptably low among the three ethnic populations.

## Competing interests

The authors declare that they have no competing interests.

## Authors’ contributions

Conceived and designed the experiments: J-YL, Y-TM. Performed the experiments: J-YL, Z-XY, Y-NY. Analyzed the data: XX, XM. Contributed reagents/materials/analysis tools: FL, X-ML, B-DC. Wrote the paper: J-YL, Y-TM. All authors read and approved the final manuscript.
